# Cat Bites in Southern Mexico: Epidemiological Patterns and One Health Perspective

**DOI:** 10.1155/vmi/6825008

**Published:** 2026-06-22

**Authors:** Ali Novelo-Sanguino, Matilde Jimenez-Coello, Daly Martinez-Ortiz, Eduardo Gutierrez-Blanco, Antonio Ortega-Pacheco

**Affiliations:** ^1^ Department of Animal Health and Preventive Medicine, Faculty of Veterinary Medicine and Animal Science, Autonomous University of Yucatan, Merida, Yucatan, Mexico, uady.mx; ^2^ Laboratory of Microbiology, CIR “Hideyo Noguchi”, Autonomous University of Yucatan, Merida, Yucatan, Mexico, uady.mx; ^3^ Department of Zoonoses, Yucatan State Ministry of Health, Merida, Yucatan, Mexico

## Abstract

There is currently limited epidemiological information about cat aggressions in Mexico. We analyzed the data of cat attacks on humans recorded between 2018 and 2023 in people attending health services in the state of Yucatan. A retrospective study was performed based on a database provided by the Yucatan Ministry of Health (SSY), which included reports of animal bites to people. Sociodemographic variables and event characteristics were selected and standardized for analysis. A total of 699 cat attacks were recorded out of 8845 animal bite reports (7.9%) during the studied period. The municipality of Merida had a higher number of reports than the rest of the municipalities in the state (*p* < 0.001). The majority of those attacked were women and adults. The median age of the attacked women was significantly higher than that of men (35 vs. 30 years; *p* < 0.001). The upper extremity was the most frequently affected anatomical area. Cat aggressions in Yucatan have shown an increasing trend, primarily affecting adult women with low post‐exposure prophylactic rabies vaccination in cats. These findings are important for the development of future preventive and epidemiological surveillance strategies within the framework of the “One Health” approach.

## 1. Introduction

The global population of domestic cats is estimated at approximately 600 million individuals [1], highlighting their popularity as companion animals. This popularity has been attributed to their affectionate nature and their effectiveness in controlling unwanted fauna in and around households. According to the World Health Organization, cat bites represent between 2% and 50% of all animal bites recorded in humans [2]. Feline aggression has been documented to be between 12% and 47% of behavioral problems reported in these animals [3] making a public health problem given their ability to cause injuries such as bites, abrasions, tears, and avulsions [4–6]. Cat bites can facilitate deep inoculation of pathogens, which leads to 20%–80% of infections, representing a higher risk compared to dog bites [2, 7]. Likewise, cats have been reported to be capable of transmitting around 40 zoonotic diseases to humans [8]. Among the most relevant zoonoses transmitted through an injury are pasteurellosis (*Pasteurella multocida*), cat scratch disease produced by *Bartonella henselae* [7, 9], and rabies, which is lethal in 100% of cases [10, 11].

Cat bites and scratches produce serious infections that may require specialized medical attention, including emergency room visits, antibiotic treatment, reconstructive surgery, and physical rehabilitation [12, 13]. In the United States, cat bites are estimated to result in approximately 300,000 emergency room visits annually [7]. These attacks occur most frequently in adult women and children under the age of 15, with the extremities being the most commonly affected anatomical regions [3].

Despite the clinical and epidemiological relevance of cat attacks, few studies documenting this problem have been conducted on this species in Mexico. Understanding the landscape of cat bites on people in the region would allow for the establishment of more effective prevention strategies aimed at mitigating the risk these attacks pose to public health. Therefore, the objective of this study was to characterize and determine the frequency of cat attacks recorded between 2018 and 2023 among people who sought healthcare services in the state of Yucatan (Southern Mexico).

## 2. Materials and Methods

### 2.1. Study Area

This research was performed in the Yucatan state, located on the southeast of Mexico. Merida is the capital city of Yucatan state (19°30′ and 21°35′ north latitude; 87°30′ and 90°24′ west longitude) with a population of about 1,250,000 inhabitants, whereas in the state of Yucatan, an approximate population of 2,498,676 inhabitants is estimated to be distributed across 106 municipalities [14].

### 2.2. Data Collection

An observational, descriptive, longitudinal, and retrospective study was conducted based on the analysis of a database provided by the Yucatan State Health Secretariat (SSY), containing records of animal attacks on people treated in the state’s health units, between January 2018 and December 2023. In the present study, only records corresponding to cat‐related attacks were filtered, from the total reported bites.

### 2.3. Processing of Data

The database processing was carried out in three stages:

1. Initial filtering of records: In this stage, only records of cat attacks were selected, excluding other reported species.
2. Data curation: Those records that presented incomplete information on any of the variables of interest were identified and excluded from the statistical analysis.
3. Selection and standardization of variables: Seven variables of interest were selected related to the sociodemographic characteristics of the person attacked, the characteristics of the event, and the attacking animal. The values for each variable were standardized into a single format for analysis. Variables considered were as follows: year (2018–2023), gender of attacked person (male/female), age, population group (child—< 12 years, teenager—13–19 years, adult—20–64 years, and senior—≥ 65 years), number of bites (single/multiple), anatomical location of bite (arms, legs, head/neck, and abdomen/thorax), status of the animal (domiciliated/free roaming), and post‐exposure prophylaxis (yes/no).

### 2.4. Statistical Analysis

Data were imported into the statistical program JASP Version 0.19.1 (Apple Silicon) to perform the corresponding statistical tests, ensuring the exclusion of categories without data. Qualitative variables (age group, sex of the attacked person, anatomical location of the injury, cat status, number of bites, and post‐exposure prophylaxis) were expressed as absolute and relative frequencies. To calculate the annual bite rate, the total number of bites recorded during the period (2018–2023) was divided by the number of years analyzed and by the human population considered (2,320,898) [14]. The result was multiplied by 100,000 to express the rate in terms of the number of people bitten per 100,000 inhabitants. To identify possible linear trends in the distribution of variables over time (2018–2023), such as the frequency of bites per year, the chi‐squared statistical test for trend was used. To evaluate associations between dichotomous categorical variables, such as the sex of the person attacked (male or female) and the status of the cat (domestic or stray), the classic chi‐squared statistical test was used. On the other hand, to compare the age between men and women attacked by cats during the study period, normality of the data was first assessed; if this assumption was not met, Student’s t‐test was considered; otherwise, the nonparametric Mann–Whitney *U* test was used. A *p* value < 0.05 was considered statistically significant.

## 3. Results

### 3.1. Prevalence

Cat attacks on humans represented 7.9% of the total incidents recorded by animals toward humans in the state of Yucatan during the period of 2018–2023 (Table 1). It should be noted that cases of cat aggressions are within a range of 6.1%–8.8%, with a tendency to increase through the years.

**TABLE 1:** Annual distribution of bites from cats and other species to humans in the state of Yucatan (2018–2023).

Year	Cats *n* (%)	Other species *n* (%)	Total
2018	128 (8.3)	1412 (91.7)	1540
2019	115 (8.8)	1191 (91.2)	1306
2020	70 (6.8)	961 (93.2)	1031
2021	100 (7.1)	1315 (92.9)	1415
2022	111 (6.1)	1719 (93.9)	1830
2023	175 (7.6)	2138 (92.4)	2313
Total	699 (7.9)	8146 (92.1)	8845

Of the total of 8845 reports of animal attacks on humans registered by the health services of the state of Yucatan during the period of 2018–2023, 699 corresponded specifically to cat attacks on humans, which represents an annual rate of 5.02 people bitten per 100,000 inhabitants.

The frequency of cat attacks by health jurisdiction of the state of Yucatan (Merida, Valladolid, and Ticul) and by municipality within each jurisdiction is shown in Table 2. The capital city, Merida, registered the greatest number of cases, and the bigger the city, the more frequent the reported cases.

**TABLE 2:** Distribution of cat attacks on humans by health jurisdiction and municipality in the state of Yucatan during the period of 2018–2023.

**Jurisdiction**	**Municipality**	***n***	

Merida	Merida	387	496
	Progreso	29	
	Kanasin	26	
	Uman	19	
	Hunucma	9	
	Motul	8	
	Dzoncahuich	3	
	Kantunil	3	
	Yobain	3	
	Others	9	

Valladolid	Valladolid	60	123
	Tizimin	16	
	Yaxcaba	8	
	Chemax	6	
	Buctzotz	5	
	Chichimila	4	
	Temozon	4	
	Tinum	4	
	Rio Lagartos	3	
	San Felipe	3	
	Uayma	2	
	Others	8	

Ticul	Ticul	38	80
	Tekax	23	
	Peto	10	
	Tzucacab	5	
	Others	4	
Total			699

**FIGURE 1:**
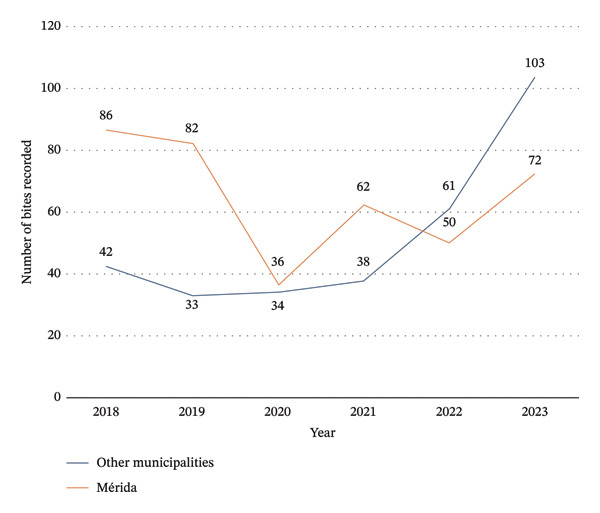
Frequency of cat bites to humans in Yucatan: differences between Merida and other municipalities (2018–2023).

The distribution of cases is presented in Figure 1. The municipality of Merida recorded a higher frequency of reports during the first 4 years compared to the rest of the municipalities in the state (*p* < 0.001) over the 6 years evaluated. During this period, Merida accounted for between 41.1% and 71.3% of the total cases of cat attacks on humans in the state. However, in the last 2 years of the study, a maintained increase in cases was observed in other municipalities. Notably, during the pandemic of COVID‐19, a light decrease of cases was observed in the city and after an increased tendency was observed.

### 3.2. Characteristics of People Attacked

No significant differences were observed in relation to sex, age group, or anatomical location of the attack (*p* > 0.05). However, a significant difference was identified regarding the administration of prophylactic treatment after the attack, with a higher proportion of people not receiving it (*p*0.037) (Table 3).

**TABLE 3:** Distribution of the characteristics of 699 people attacked by cats in the state of Yucatán during the period of 2018–2023.

Year	Sex *n* (%)		Age group *n* (%)				Location of the bite *n* (%)				Prophylaxis^∗^ *n* (%)	
	Women	Men	Child	Teenager	Adult	Senior citizen	Arms	Legs	Head/neck	Abdomen/thorax	Yes	Not
2018	90 (70)	38 (30)	17 (13)	18 (14)	75 (59)	18 (14)	81 (63)	45 (35)	1 (1)	1 (1)	37 (29)	91 (71)
2019	66 (57)	49 (43)	13 (12)	21 (18)	67 (58)	14 (12)	62 (54)	51 (44)	0	2 (2)	39 (34)	76 (66)
2020	46 (66)	24 (34)	7 (10)	12 (17)	39 (56)	12 (17)	39 (56)	31 (44)	0	0	21 (30)	49 (70)
2021	59 (59)	41 (41)	9 (9)	19 (19)	61 (61)	11 (11)	68 (69)	27 (28)	0	3 (3)	48 (48)	52 (52)
2022	68 (61)	43 (39)	16 (14)	19 (17)	56 (51)	20 (18)	57 (51)	52 (47)	1 (1)	1 (1)	38 (34)	73 (66)
2023	115 (66)	60 (34)	21 (12)	32 (19)	104 (59)	18 (11)	98 (56)	72 (41)	4 (2)	1 (1)	53 (30)	122 (70)
Total	444 (64)^a^	255 (36)^a^	83 (12)^a^	121 (17)^a^	402 (58)^a^	93 (13)^a^	405 (58)^a^	278 (40)^a^	6 (1)^a^	8 (1)^a^	236 (34)^a^	463 (66)^a^

*Note:* Data are presented as absolute and relative frequencies. Statistical analysis was performed using the *X* ^2^ trend test. Two cases were excluded in which the anatomical location of the aggression was not specified.

^a^Percentage calculated based on the total number of cases reported in the period 2018–2023.

^∗^A difference was considered statistically significant when *p* < 0.05.

Although owned cats accounted for a higher proportion of bite incidents compared to free‐roaming cats (65% vs 35%), no significant trend over time was observed (*χ* ^2^ trend test, *p* > 0.05). However, when evaluating the overall distribution, a significant difference was found between categories (*χ* ^2^ = 57.6, df = 1, *p* < 0.001). These findings reflect the distribution of reported cases and should be interpreted with caution, as the underlying population of cats was not assessed (Table 4). Thirty‐one records were excluded due to lack of information on the status of the aggressor cat and four records due to failure to specify the number of times the cat bit.

**TABLE 4:** Distribution of the characteristics of the aggressive cat in the state of Yucatán during the period of 2018–2023.

Year	Status^∗∗∗^ *n* = 668 (%)		Number of bites^∗∗^ *n* = 695 (%)	
	Domiciled	Free roaming	Single	Multiple
2018	81 (73)	30 (27)	106 (83)	22 (17)
2019	54 (53)	48 (47)	99 (86)	16 (14)
2020	50 (71)	20 (29)	58 (83)	12 (17)
2021	60 (60)	40 (40)	67 (70)	29 (30)
2022	71 (65)	39 (35)	78 (70)	33 (30)
2023	116 (66)	59 (34)	133 (76)	42 (24)
Total	432 (65)	236 (35)	541 (78)	154 (22)

*Note:* Data are presented as absolute and relative frequencies. Statistical analysis was performed using the *X* ^2^ goodness and *X* ^2^ trend test.

^∗∗^ *p* < 0.005.

^∗∗∗^ *p* < 0.001.

**FIGURE 2:**
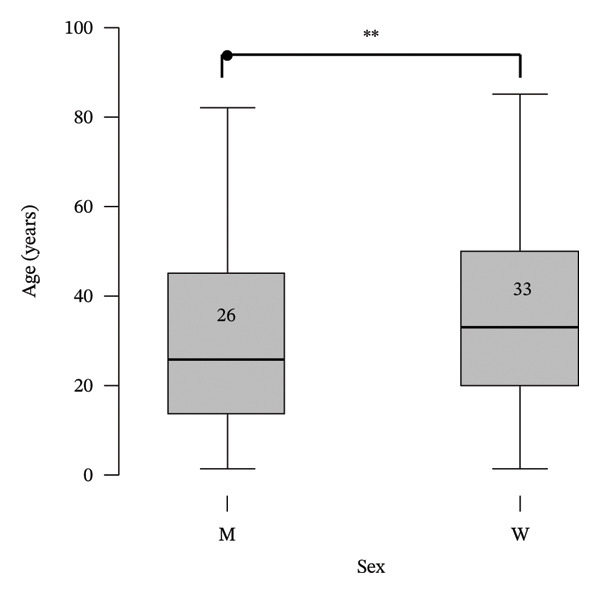
Comparison of the age of people attacked by cats by sex in the state of Yucatán, during the period of 2018–2023. ^∗∗^ *p* < 0.001. Men (M); women (W).

When comparing the age of people attacked by cats according to their sex, a total of 255 men and 444 women were included. The median age was 26 years (IQR: 14–45) for men and 33 years (IQR: 20–50) for women. As data were not normally distributed (Shapiro–Wilk test, *p* < 0.001), a Mann–Whitney *U* test was applied, revealing a statistically difference between groups (*U* = 46,427, *p* < 0.001). These results indicate that women affected by cat bites tended to be older than men. Age is presented as a continuous variable, consistent with the statistical approach used (Figure 2).

When comparing the victims with different characteristics (Table 5), the age group (*p* < 0.004) and the anatomical location of the bite (*p* < 0.0001) differed significantly by sex. Statistically significant differences were also observed between sex and the number of attacks suffered (*p* < 0.034), as well as in the proportion of people who received or did not receive prophylactic treatment (*p* < 0.021).

**TABLE 5:** Comparison of the sex of people attacked by cats with the age group, location of the attack, number of bites, and prophylactic treatment received in the period 2018–2023.

Sex of the person attacked	Age group^∗^				Location of the aggression^∗^				Number of bites^∗^		Prophylaxis for the^∗^ person attacked	
	Child	Teenager	Adult	Senior citizen	Upper limb	Lower limb	Head or neck	Abdomen/thorax	Unique	Multiple	Received	Did not receive
Women	40	70	270	64	229	207	2	6	356	87	136	308
Men	43	51	132	29	176	71	4	2	185	67	100	155
Total	83	121	402	93	405	278	6	8	541	154	236	463

*Note:* Data are presented as absolute frequencies. Statistical analysis was performed using the *X* ^2^ test and *X* ^2^ for trend. Data were excluded if the anatomical location of the aggression was not specified and if the number of cat bites on the victims was not specified.

^∗^ *p* < 0.05.

Of the 699 reports of cat aggression toward humans, only 668 contained information on the status of the aggressor cat (domestic or free roaming) (Table 6). When comparing this status with the characteristics of the attacked individuals and the attack, significant differences were observed in the sex of the attacked individual (*p* < 0.0025), the anatomical location of the bite (*p* < 0.017), and whether prophylactic treatment was received (*p* < 0.0001). No significant differences were found in the age group of the attacked individuals (*p* < 0.117) or in the number of bites (*p* < 0.606).

**TABLE 6:** Comparison of the status of the aggressor cat with the characteristics of the attacked people, the location of the attack, the number of bites, and the prophylactic treatment received in the period 2018–2023.

^∗^Cat status	Sex of the person^∗^ attacked		Age group				Location of the aggression^∗^				Number of bites		Prophylaxis for^∗^ the person attacked	
	Women	Men	Child	Teenager	Adult	Senior citizen	Upper limb	Lower limb	Head or neck	Abdomen/thorax	Single	Multiple^∗^	Yes	No
Domiciled	296	136	56	76	233	67	233	187	5	5	340	90	33	399
Street	134	102	23	41	147	25	155	79	0	2	181	53	190	46
Total	430	238	79	117	380	92	388	266	5	7	521	143	223	445

*Note:* Data are presented as absolute frequencies. Statistical analysis was performed using the *X* ^2^ test and *X* ^2^ for trend. Data in which the anatomical location of the aggression was not specified and in which the number of cat bites on the attacked people was not specified were excluded. Cases in which the status of the attacking cat was not recorded were also excluded.

^∗^ *p* < 0.05.

## 4. Discussion

To our knowledge, this is the first epidemiological study of cat bites on humans in Southern Mexico. Cats are recognized as the second most common species of bites on humans, behind dogs. Various studies have reported that cat bites represent between 5% and 10% of cases [15]. Dog bites range from 70.9% to 91.6% of all animal attacks on humans [5, 16–19].

In the present study, 7.9% of animal attacks were caused by cats, which is within the range already reported. This value is close to that reported in Valencia, Spain (8%) [16], and the United States (8.8%) [20]. Our results differ from those in India and Iran, where higher proportions were observed, with 24.3% and 21.3%, respectively [5, 21], compared to those reported in other countries. Compared to other Latin American countries, our results are slightly close to those in Chile (5.6%) [17], but below those observed in Brazil (12.5%) [18]. At national level, a study conducted in the municipality of Coyoacán reported a slightly higher frequency (9.4%) [19]. Clearly, a great variation in the prevalence of cat bites is present worldwide depending probably on the cultural practices and habits with cat keeping. Since the reports are based on cases attending to clinics or hospitals, it is also probable that this variation may also be influenced by the number of people attacked by cats attending to clinical services and registration of cases. This also may explain why more cases are reported in large cities where more medical attention clinics are present.

During the studied period, the average annual rate of cat attacks on humans in the state of Yucatan was 5.02 cases per 100,000 inhabitants. This is much lower than that reported in Brazil, where 41 bites per 100,000 people were documented between 2008 and 2016 [18], but it is higher than that recorded in Uruguay, estimated at 2.15 annual cases per 100,000 inhabitants during 2010–2020 [22]. In Spain, a slightly higher rate was reported, with an annual average of 6.36 incidents per 100,000 people [16]. These differences could be explained by demographic factors, feline population density, human–animal coexistence habits, and characteristics of the epidemiological surveillance systems existing in each country [16, 18, 22].

In the present study, when considering the general distribution of cat bites, it is observed that Merida (urban area) had the highest incidence of bites. This aligns with other studies, like in Iran, where the prevalence of bites in urban populations (76%) was approximately 1.07 times higher than that in rural areas [21], or Brazil, where 84.3% of bite reports came from urban areas and only 10% from rural areas [18]. The higher population density of cats in cities and better access of attacked persons to health services may favor the reporting of cases and the apparently higher frequency of bites.

Contrary to what occurred with dogs, the number of attacks caused by cats during the COVID‐19 pandemic decreased significantly, as found in Uruguay [23] and in other regions of Mexico [19]. It is reported that a large number of pets were relinquished during the pandemic, apparently due to financial problems, health problems associated with COVID‐19, and behavioral problems [24], and thus less aggressions occurred. However, it is more likely that the during the confinement, visits to the clinics after an aggression was evade, which may be also a factor that could be associated with a lower number of reported cases.

Only 34% of people bitten by a cat received prophylactic treatment; a similar proportion to that reported in a municipality in Mexico City was reported [19]. Furthermore, the significant difference between those who received and those who did not found in the present study highlights a possible omission in post‐exposure care, which not only increases the risk of local infections but also, in extreme situations, could endanger the lives of the victims, especially when cat rabies is considered a global emerging public health issue [25], with fatal cases reported particularly in children [26]. Post‐bite complications such as cat scratch disease, cellulitis, necrotizing fasciitis, tetanus, abscess, and transmission of other viruses may also occur [27, 28]. However, in the present study, no data about post‐bite complications other than rabies were included in the registers; such information would be usefully to be considered for a better epidemiological picture of cat bites.

In the present study, older people, particularly women, were more prone to cat bites as seen in previous reports; for example, in a study conducted in Coyoacán, Mexico, the mean age of people with more cat aggressions was 32 years for both sexes [19]; in India and Iran, a mean of 31.0 and 32.7 years was reported, respectively [5, 21]. These findings suggest that the adult population, particularly women, could represent a group with greater exposure to bite attacks, possibly associated with their caregiver role and management of pets including tasks such as feeding, cleaning, medical care, or rescuing stray cats, which increases their exposure to the risk of being bitten [29]. In Uruguay, adult women were also the most affected group, accounting for 66.5% of cat bites recorded between 2010 and 2020 [22]. Similarly, in Spain, both adult women and minors were the groups most likely to suffer this type of aggression [16], supporting the trend observed in the present study. Furthermore, some attacks may be linked to attempts to handle or restrain animals during episodes of stress or illness [29].

As previously reported [16, 21, 30], single bites are more common in cat aggressions particularly in hands, wrists, and arms. In Valencia, Spain, 86% of the wounds were single, puncture‐type, and predominantly in the upper extremities. These aggressions are probably due to attempts of people to manipulate, feed, or physically intervene in the behavior of the aggressor animal [28].

## 5. Conclusions

This study documents the epidemiology of cat bites to humans in the state of Yucatan, demonstrating a sustained increase in cases between 2018 and 2023 and highlighting the relevance of this type of injury as a public health problem. The predominance of older adult female victims, the frequent involvement of the upper extremities, and the high proportion of cases without post‐exposure prophylaxis point out the need to strengthen preventive strategies and ensure timely clinical intervention. The participation of both owned and free‐roaming cats, coupled with their potential to transmit zoonotic pathogens, emphasizes the importance of incorporating this species into epidemiological surveillance systems, population control programs, and public education initiatives on responsible ownership. Addressing this issue from a comprehensive One Health perspective will not only reduce the incidence of cat bites but also contribute to mitigating the risk of transmission of infectious diseases, thus protecting human, animal, and environmental health in the region.

## Author Contributions

Antonio Ortega‐Pacheco and Matilde Jimenez‐Coello were responsible for conceptualization, funding acquisition, data analysis, result discussion and interpretation, visualization, writing–original draft, and writing–review and editing. Ali Novelo‐Sanguino, Eduardo Gutierrez‐Blanco, and Daly Martinez‐Ortiz analyzed the data, interpreted and discussed the results, and reviewed and edited the manuscript. Eduardo Gutierrez‐Blanco and Ali Novelo‐Sanguino designed and performed the experiments, analyzed the data, and reviewed and edited the manuscript.

## Funding

No funding was received for this manuscript.

## Disclosure

All authors have read and approved the final manuscript.

## Conflicts of Interest

The authors declare no conflicts of interest.

## Data Availability

All data pertaining to the current study are available from the corresponding author upon reasonable request.
